# Unveiling the landscape of plant virology in Saudi Arabia: seven decades of progress and future directions toward Vision 2030

**DOI:** 10.3389/fpls.2026.1758142

**Published:** 2026-04-10

**Authors:** Zafar Iqbal, Aneesa Zeb Awan, Sidra Atta, Khadim Hussain, Muhammad Khurshid, Fiaz Ahmad, Muhammad Munir, Abdul Ghafoor, Sallah A. Al Hashedi, Khaled M. A. Ramadan, Sherif Mohamed El-Gananiny, Mohammed Ali AlSaleh

**Affiliations:** 1 Central Laboratories, King Faisal University, Al Ahsa, Saudi Arabia; 2 School of Biochemistry and Biotechnology, University of the Punjab, Lahore, Pakistan; 3 Plant Protection Department, College of Food and Agriculture Sciences, King Saud University, Riyadh, Saudi Arabia; 4 Key Laboratory for Space Bioscience & Biotechnology, School of Life Sciences, Northwestern Polytechnical University, Xi’an, China; 5 Date Palm Research Center of Excellence, King Faisal University, Al Ahsa, Saudi Arabia; 6 Center for Water and Environmental Studies, King Faisal University, Al Ahsa, Saudi Arabia; 7 Department of Arid Land Agriculture, College of Agriculture and Food Sciences, King Faisal University, Al Ahsa, Saudi Arabia

**Keywords:** CRISPR-Cas, plant virology, plant viruses, Saudi Arabia, sustainability, virus detection, Vision 2030

## Abstract

Plant viruses pose a persistent and escalating threat to global agriculture and food security, inflicting over $30 billion in annual losses – a challenge acutely felt in Saudi Arabia as it strives for agricultural self-sufficiency under Vision 2030. This is the first comprehensive review which presents seven decades of plant virology research in the Kingdom, from early symptom-based diagnosis to advanced molecular, genomic, and bioinformatics advances. A total of ~81 plant viral species infecting 46 plant host species have been documented across the major agroecological regions, dominated by positive-sense single-stranded RNA viruses (~70%). Among these viruses, some are economically most destructive—including alfalfa mosaic virus, cucumber mosaic virus, soilborne cereal mosaic virus, tomato yellow leaf curl virus, zucchini yellow mosaic virus, watermelon chlorotic stunt virus, and barley mild mosaic virus—posing recurrent challenges to key crops such as alfalfa, cucurbits, and tomatoes. Network analysis of virus distribution revealed strong epidemiological linkages among central and western agricultural regions, possibly driven by intensive cultivation and vector ecology. The review highlights emerging management strategies including CRISPR–Cas diagnostics, RNA interference, AI-based detection, nanotechnology, and plant growth promoting rhizobacteria. Gaps persist in genomic surveillance, vector ecology, and biosecurity enforcement. The review concludes with future research priorities emphasizing innovation, interdisciplinary collaboration, and the development of a national plant virus genomic and surveillance framework to secure sustainable agriculture in line with Vision 2030.

## 1 Introduction

**Figure 1:**
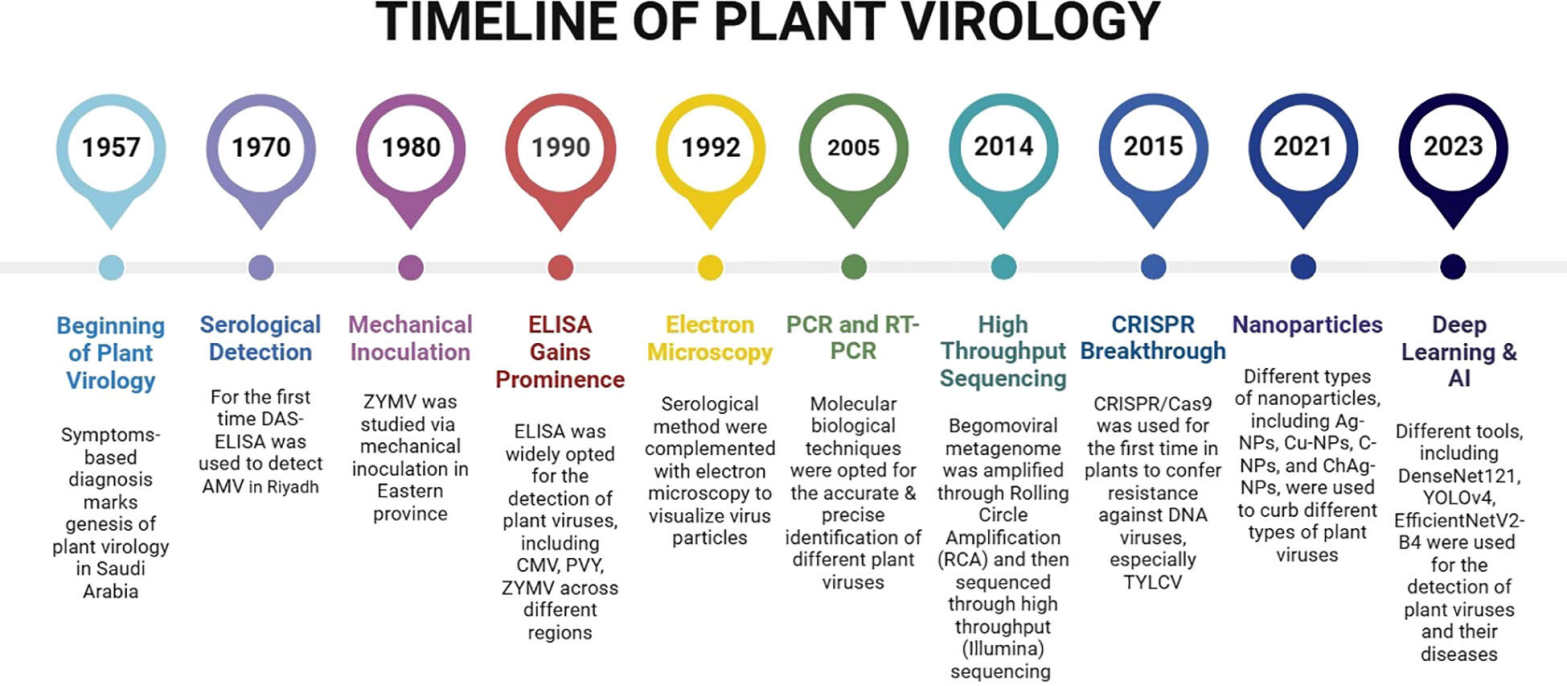
Evolution of plant virology in Saudi Arabia: from symptom-based diagnosis to molecular innovations (1957–2025).

Plant viruses are one of the most severe and persistent threats to global agriculture, causing over US$ 30 billion in direct annual losses (Savary et al., 2019; Tatineni and Hein, 2023). Currently, over 2,025 virus species from 73 genera and 49 families are recognized (https://ictv.global/vmr). Their efficient transmission—via insect vectors such as aphids, whiteflies, and thrips, as well as through seeds, mechanical contact, and human-mediated trade—enables rapid dissemination across regions and borders. Such transmission pathways enabled rapid dissemination across regions and national borders, allowing localized outbreaks to global agricultural crises. Saudi Arabia’s agriculture spans arid and semiarid ecosystems and is particularly vulnerable to viral outbreaks. Intensive cultivation, heavy reliance on imported planting material, and climatic stress increase this vulnerability. Since the first visual diagnosis of tomato mosaic like symptoms in 1957 in the Eastern Province (Talhouk, 1957), plant virology in Saudi Arabia has evolved through four distinct phases: (i) symptom based diagnostics (1950s–1980s), (ii) serological assays (1980s–2000s), (iii) molecular detection (2000s–2010s), and (iv) high-throughput sequencing (HTS) and Clustered regularly interspaced short palindromic repeats (CRISPR)-CRISPR associated protein (Cas) based interventions (2010s–present) (**Figure 1**).

Despite recent progress, plant virology in Saudi Arabia remains an emerging discipline, hindered by. Notably, the application of modern bioinformatic approaches have revealed that Watermelon chlorotic stunt virus (WmCSV) has been endemic to the region for centuries, establishing Saudi Arabia as a major center of viral diversity and a key hub for its dissemination.

Despite some advances, Saudi plant virology remains in early development, constrained by persistent limitations in research capacity and infrastructure. It is noteworthy to mention that recent adoption of bioinformatics approaches have revealed that watermelon chlorotic stunt virus (WmCSV) has been endemic to the region for centuries, establishing Saudi Arabia as a major center of viral diversity and a key hub for its dissemination (Iqbal, 2025a). So, it is apparently important to note that the identification of new plant viruses is driven both by diagnostic evolution and might reflect epidemiological turnover.

Saudi Arabia’s Vision 2030 provides a strategic context in which agricultural expansion, technological intensification, economic diversification, sustainability, and food security goals intersect with plant health risks. While intensified farming and technological innovation can enhance productivity, they also increase the likelihood of virus introductions, expansion of viral vectors, and more frequent outbreaks. Such scenario threatens to undermine resource-use efficiency and crop resilience, underscoring the need for evidence-based surveillance and management. Framing plant virology within the Vision 2030 agenda therefore shifts emphasis from descriptive pathogen inventories toward applied outcomes, including strengthened diagnostics, resilient production systems, and reduced dependence on chemical control. In this context, plant virology emerges as a critical component of national efforts to enhance agricultural sustainability, biosecurity, and biotechnology-driven innovation.

This review was aimed at providing a comprehensive overview of the evolution of plant virology in Saudi Arabia. It traces historical development and consolidates national efforts to map viral diversity across key crops. The review highlights advancements in diagnostics, research infrastructure, and capacity building, while identifying existing gaps and challenges in surveillance, management, and policy integration. Furthermore, the review aligns these scientific insights with the strategic goals of Saudi Vision 2030, emphasizing agricultural sustainability, biosecurity, and innovation-driven food security.

## 2 The plant viral landscape of Saudi Arabia

**Figure 2:**
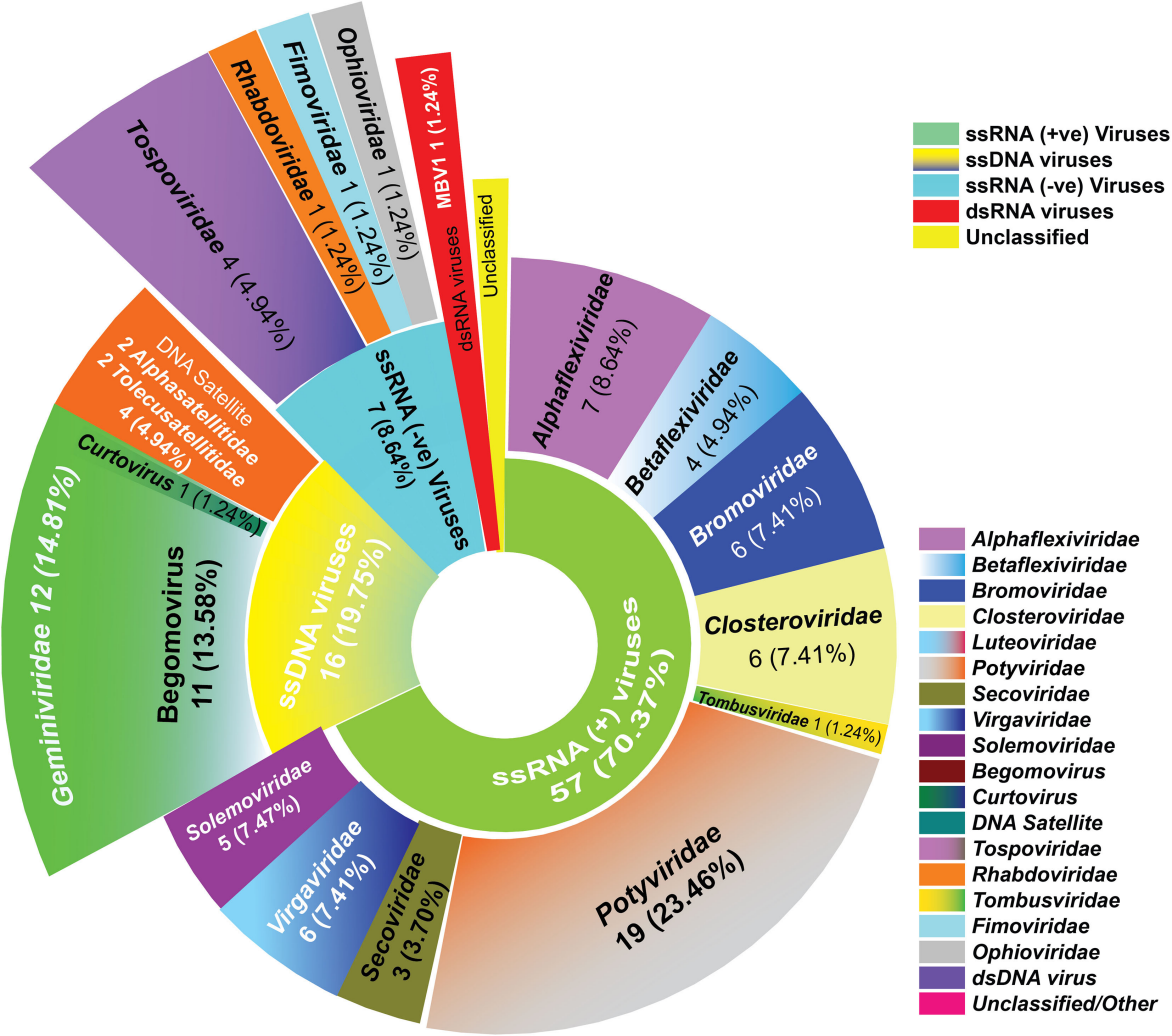
Taxonomic distribution of plant viruses in Saudi Arabia: dominance of +ssRNA viruses across all identified species. Inner circles represent classes of viruses and outer represents families of viruses. The number and percentage of virus classes and families are mentioned.

Saudi Arabia’s diverse agro-climatic zones–spanning arid, semiarid, and subtropical gradients–forms a complex tapestry of virus–host–vector interactions. Major agricultural hubs, including Riyadh, AlQassim, Hail, Tabuk, AlAhsa, Jazan, and AlJouf, serve as epicenters for viral outbreaks, driven by intensive farming and insect vector proliferation. As of 2025, Saudi Arabia’s viral landscape comprises about 81 plant viruses across four genome classes (as per ICTV Virus Metadata Resource 2024). Positive-sense single-stranded RNA (+ssRNA) viruses predominate (n=57; ~70.37%), followed by ssDNA viruses (n=16 [includes 4 DNA satellites]; ~19.75%), negative-sense ssRNA viruses (n=7; ~8.64%), and a dsDNA virus ([n=1; ~1.24%]; **Supplementary Table S1**; **Figure 2**).

The apparent dominance of +ssRNA viruses and the chronological cluster of first reports within the Kingdom must be critically evaluated as a function of advancing diagnostic resolution than purely biological emergence. Historically, surveillance in Saudi Arabia was limited by the detection limits of symptomatology and enzyme-linked immunosorbent assay (ELISA), which are inherently biased toward high-titer, symptomatic +ssRNA pathogens in staple crops (Al-Shahwan et al., 1995). The transition to polymerase chain reaction (PCR)-based diagnostics enhanced taxonomic specificity and the detection of cryptic complexes (Al-Saleh et al., 2014a). While the recent integration of HTS has started unveiling a “hidden” diversity of low-titer, latent, and multi-viral infections previously overlooked by targeted assays. Consequently, the contemporary Saudi agro-virome likely reflects a technological artifact—an “inflation” of viral richness driven by expanded diagnostic sensitivity—rather than an abrupt shift in viral ecology, which reported spatiotemporal distributions are primarily indices of surveillance intensity.

### 2.1 Positive-sense ssRNA plant viruses

This dominant viral class, 70.37% of all reported plant viruses in Saudi Arabia, spans 10 families, with *Potyviridae* the most impactful (19 species, 17 potyviruses). Other significant families include *Alphaflexiviridae* (7 species), *Bromoviridae* (6), *Closteroviridae* (6), *Virgaviridae* (6), and *Betaflexiviridae* (4), among others (**Supplementary Table S1**; **Figure 2**).

These viruses are widely distributed, especially in Riyadh, AlQassim, Hail, Tabuk, AlAhsa, and AlJouf. Riyadh has the longest surveillance record, from alfalfa mosaic virus (AMV) detection in 1971 (Abu-Yaman and Abu-Blan, 1973), to subsequent viruses detections (Al-Shahwan, 2002; Al-Saleh and Amer, 2013; Al-Saleh et al., 2014c; Alhudaib, 2019; Abdalla et al., 2020), and tomato brown rugose fruit virus (ToBRFV, 2021–2022) (Sabra et al., 2022). AlQassim is another hotspot, notably for potato virus X (PVX; Al-Shahwan et al., 1997), pepino mosaic virus (Sabra et al., 2021), and garlic viruses A–C (GarV A–C; Amir et al., 2024). Hail and Tabuk have also been frequently surveyed, with higher occurrence of potato virus S (Al-Shahwan et al., 1997) and cucumber mosaic virus (CMV) (Salama et al., 1987). Other regions, including AlBaha, AlAhsa, and Western Saudi Arabia (Makkah and Jeddah), showed more sporadic occurrences, often linked to specific crops like figs (fig leaf mottle-associated viruses; Aldhebiani et al., 2015) or periwinkle (catharanthus mosaic virus [CatMV]; Elbeshehy et al., 2017). The temporal range of detections, from 1971 AMV to 2025 (garlic viruses), underscores the persistence and emergence of +ssRNA viruses, emphasizing the need for continued surveillance and research.

The host range of +ssRNA viruses is highly diverse, encompassing staple and cash crops plus weeds. Potato (*Solanum tuberosum*) is infected by PVX, potato virus Y (PVY), potato virus M (PVM), and potato leaf roll virus (PLRV, a polerovirus). Cucurbits (cucumber, watermelon, melon) host zucchini yellow mosaic virus (ZYMV), cucumber green mottle mosaic virus (CGMMV), and watermelon mosaic virus (WMV), while alfalfa (*Medicago sativa* L.) is affected by AMV, pea streak virus (PeSV), and red clover vein mosaic virus (RCVMV). Garlic and onion harbor GarV A–D and onion yellow dwarf virus (OYDV), and capsicum is affected by pepper mild mottle virus (PMMoV) and pepper mottle virus (PepMoV) (**Supplementary Table S1**). Weeds like *Sonchus oleraceus*, *Chenopodium* spp., and *Malva parviflora* act as reservoirs, facilitating cross-species transmission and economic losses. Detection approaches encompass ELISA (for PVX, AMV), RT-PCR (for PVY, FLMaV1), agar diffusion test, electron microscopy, and next-generation sequencing (for PLRV).

### 2.2 Negative-sense ssRNA plant viruses

Saudi Arabia’s viral landscape includes seven −ssRNA viruses in four families, including *Tospoviridae* (4 species), *Rhabdoviridae* (1), *Fimoviridae* (1), and *Ophioviridae* (1). Fig mosaic virus (FMV; family *Fimoviridae*), first identified in 2015 in West Makkah, infects fig trees (*Ficus carica*) and was detected via RT-PCR (Aldhebiani et al., 2015). The *Tospoviridae* family (genus *Orthotospovirus*) includes tomato spotted wilt orthotospovirus (TSWV), a highly destructive pathogen first reported in AlQassim and Riyadh in early 1990s, with later detections in Hail, Tabuk, AlHofuf, Najran, AlUyaynah, and Taif using ELISA and RT-PCR. This persistent and widespread detection from tomato (*Solanum lycopersicum*), lettuce, and periwinkle (*Catharanthus roseus* L.), reflects persistent occurrence. Three other orthotospoviruses, including capsicum chlorosis orthotospovirus (CaCV), Tomato yellow ring orthotospovirus (TYRV), and tomato chlorotic spot orthotospovirus (TCSV), were detected in the AlQassim, AlBaha, Riyadh, and AlKharj (2020-2022) on peppers (*Capsicum annuum* L.), lettuce, and tomato using DAS-ELISA (Khalid et al., 2024; Al-Shudifat et al., 2021). The *Rhabdoviridae* family (genus *Varicosavirus*) includes lettuce big vein associated varicosavirus (LBVaV), identified in Riyadh (2014-2015) from lettuce (*Lactuca sativa* L.) via DAS-ELISA and RT-PCR (Umar et al., 2017).

### 2.3 ssDNA plant viruses

In Saudi Arabia, sixteen ssDNA pathogens have been identified infecting economically important crops. These include eleven begomoviruses (family *Geminiviridae*), one curtovirus (family *Geminiviridae*) and their four associated DNA satellites, two betasatellite (family *Tolecusatellitidae*) and two alphasatellite (family *Alphasatellitidae*). Tomato yellow leaf curl virus (TYLCV), reported since the 1950s, remains the most studied, infecting tomatoes and *Mentha longifolia* across regions such as Jeddah, Makkah, Tabuk, and Hail (Talhouk, 1957; Mazyad et al., 1979). Detection methods have evolved from visual diagnosis (Abdou, 1973) to ELISA (Al-Shahwan et al., 1997), PCR (Ajlan et al., 2007), and HTS (Sattar et al., 2024). Additional viruses—tomato leaf curl Palampur virus and tomato leaf curl Oman virus (ToLCOMV)—infect tomatoes, muskmelons, cucumbers, and chili peppers in AlAhsa and Jeddah (Sohrab, 2020; Sattar et al., 2024).

WmCSV infects watermelon, zucchini, cucumber, and papaya in Jeddah, AlLith, Jazan, and AlHofuf (Al-Saleh et al., 2014a; Ahmad et al., 2018; Alhudaib et al., 2018; Amer et al., 2024). Potato yellow mosaic virus (PYMV) was identified in *C. annuum* from AlQassim (Khalid et al., 2024), while bean dwarf mosaic virus (BDMV) appeared in *Phaseolus vulgaris* from AlAhsa (Ghanem et al., 2003; Sohrab, 2020). Other notable reports include catharanthus yellow mosaic virus (CaYMV) in periwinkle (Elbeshehy et al., 2017), cotton leaf curl Gezira virus (CLCuGeV) in tomato and muskmelon (Sattar et al., 2024), and okra leaf curl virus (OLCV) in okra, often linked with okra yellow crinkle Cameroon betasatellite and okra leaf curl Oman betasatellite, belonging to genus *Betasatellite* (family *Tolecusatellitidae*). Additionally, another DNA satellite, okra yellow crinkle Cameroon alphasatellite (genus *Gosmusatellite*; family *Alphasatellitidae*) and tomato yellow leaf curl betasatellites have been reported from Saudi Arabia (Sohrab, 2020; Sattar et al., 2024).

### 2.4 Double-stranded DNA plant viruses

Double-stranded DNA (dsDNA) plant viruses are comparatively rare but include important pathogens of the family *Caulimoviridae*, mainly the genera *Caulimovirus* and *Badnavirus* (Teycheney et al., 2020). In Saudi Arabia, mulberry badnavirus 1 has been detected through partial genome sequencing, though its impact on mulberry cultivation remains unclear.

### 2.5 dsRNA plant viruses

No dsRNA plant viruses (family *Reoviridae*) have been reported in Saudi Arabia to date. Globally, reoviruses infect numerous crops, are transmitted persistently by insects, and contain segmented genomes encoding one or two proteins per segment.

## 3 Economically important and emerging plant viruses in Saudi Arabia

**Figure 3:**
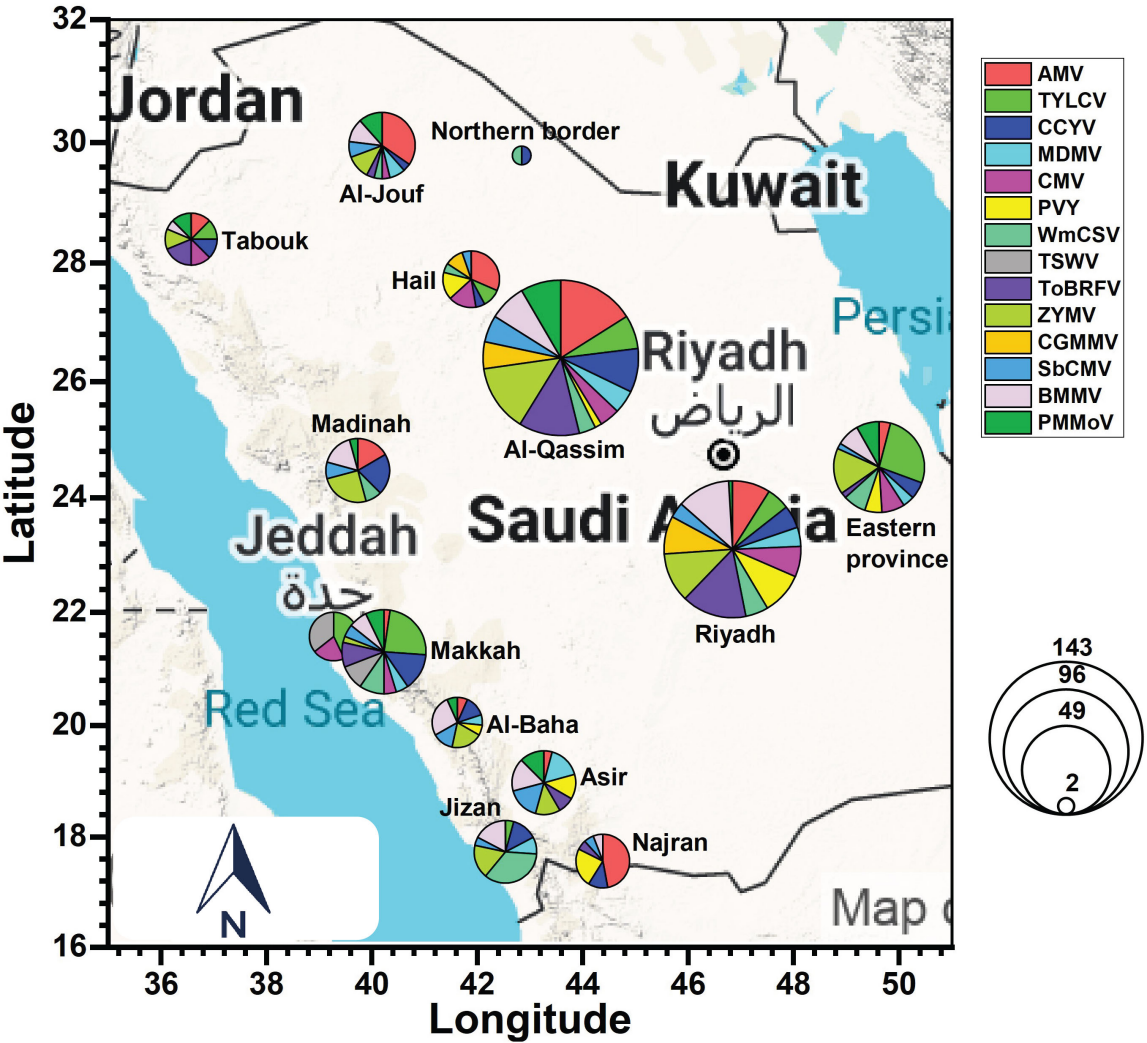
Geographic distribution of key plant viruses in Saudi Arabia, highlighting regional agricultural vulnerabilities. Graph was generated in Origin Pro v2024.

This section describes economically important and emerging plant viruses in Saudi Arabia (**Table 1**; **Figure 3**). These viruses induce severe symptoms, including leaf (yellowing, curling) and fruit (irregular shape, spots) deformities, and stunted development. Spatial analysis (**Figure 3**) reveals pronounced regional heterogeneity; high-intensity agricultural hubs—Riyadh, AlQassim, Eastern Province, and Tabuk—exhibit higher virus population and broader taxonomic representation. This distribution partly reflects intensified surveillance and year-round production—all increasing virus introduction and persistence opportunities. Conversely, lower diversity in peripheral regions likely reflects reduced cropping intensity, harsher agroclimatic conditions, and underrepresentation in surveillance rather than true epidemiological absence.

**Table 1:** Most prevalent and destructive plant viruses in Saudi Arabia: hosts, transmission mode, yield losses, and regional distribution.

Sr. no.	Virus name	Vial genus & family	Host plants	Transmission mode	Symptoms	Reported yield losses	Regions
1	Alfalfa mosaic virus (AMV)	*Alfamovirus*, *Bromoviridae*	alfalfa, tobacco, potato, *Sonchus oleraceus, Solanum tuberosum, Chenopodium quinoa, Convolvulus arvensis, Malva parviflora*, *Hibiscus* spp.*, Hippuris vulgaris, Cichorium intybus, Flaveria trinervia, Vigna unguiculata, Capsicum annuum, S. melongena, Rubus fruticosus*	aphids and seed	mosaic patterns, mottling, and stunting	Up to 58%	AlQassim, Eastern province (AlAhsa, AlHofuf, Abqaiq, AlOyun), Tabuk, AlJouf, Riyadh, Hail, Makkah, Asir, AlBaha, Madinah, Najran
2	Cucumber mosaic virus (CMV)	*Cucumovirus*, *Bromoviridae*	potato, alfalfa, *S. oleraceus, V. unguiculata, Hibiscus* spp.*, Cucurbita maxima, Chenopodium amaranticolor*, cucumber, tomato, pepper, watermelon, periwinkle,	aphids, seeds, sap, mechanical contact	mosaic patterns, stunted growth, and malformed fruit	Up to 50%	AlQassim, Riyadh, Tabuk, Hail, AlJouf, Eastern province (AlAhsa, AlHofuf, Abqaiq, AlOyun), Makkah, Jeddah
3	Zucchini yellow mosaic virus (ZYMV)	*Potyvirus*, *Potyviridae*	cucumber, squash, melon, watermelon, luffa, cucurbits, pumpkin	aphids, seeds, and sap	Leaf yellowing, filiform (shoestring) leaves, dark green blisters, and deformed fruit	Up to 80%	Riyadh, AlQassim, Eastern province (AlAhsa, AlHofuf, Abqaiq, AlOyun), Asir, AlJouf, Jazan, Makkah, Najran, Tabuk, Baha, Madinah
4	Tomato yellow leaf curl virus (TYLCV)	*Begomovirus*, *Geminiviridae*	tomato, *Phaseolus vulgaris* L., mentha (*Mentha Longifolia*), cucumber, chilies, ridge gourds	whitefly (*B. tabaci*)	leaf yellowing and curling, and stunting	50-70%	Jeddah, Makkah, Tabuk, Jazan, Hadasham, AlQassim, Jeddah, Hail, Eastern province (AlAhsa, AlHofuf, Abqaiq, AlOyun),
5	Maize dwarf mosaic virus (MDMV)	*Potyvirus*, *Potyviridae*	maize, sorghum, barley, alfalfa, blue panic (*Panicum antidotale*) grass, rhodes grass (*Chloris gayana*)	aphids	stunting, mosaic patterns, and chlorosis	Up to 90%	AlJouf, AlQassim, Jazan, Eastern province (AlAhsa, AlHofuf, Abqaiq, AlOyun), Makkah, Riyadh, Asir, Baha, Madinah
6	Cucurbit chlorotic yellows virus (CCYV)	*Crinivirus*, *Closteroviridae*	cucumber, squash, melon, watermelon	whitefly (*B. tabaci*)	leaf yellowing, stunting, and deformed fruit	Up to 62%	Riyadh, AlQassim, Hail, Eastern province (AlAhsa, AlHofuf, Abqaiq, Al-Oyun), Makkah, Northern Border, Madinah, Jazan, AlJouf, Najran, Tabuk, Baha
7	Soil-borne cereal mosaic virus (SBCMV)	*Furovirus*, *Virgaviridae*	maize, sorghum, millet, sugarcane, blue panic (*Panicum antidotale*) grass, rhodes grass (*C. gayana*), barley, wheat	protists (*P. graminis*)	growth stunting, chlorotic mosaics, and reduced tillering	20–50%	AlJouf, AlQassim, Jazan, Riyadh, Hail, Makkah, Najran, Eastern province (AlAhsa, AlHofuf), Asir, Baha, Madinah
8	Watermelon chlorotic stunt virus (WmCSV)	*Begomovirus*, *Geminiviridae*	cucumber, squash, melon, watermelon, papaya, zucchini	whitefly (*B. tabaci*)	leaf chlorosis, yellow mosaic, curling, and fruit deformities	50-90%	Riyadh, Eastern province (AlAhsa, AlHofuf, Abqaiq, AlOyun), AlQassim, Jazan, Makkah, AlJouf, Jazan, Hail, Northern Border, Madinah
9	Barley mild mosaic virus (BaMMV)	*Bymovirus*, *Potyviridae*	maize, Sorghum, Blue panic (*P. antidotale*) grass, Rhodes grass (*C. gayana*), Barley, Wheat	protists (*P. graminis*	leaf discoloration, mosaic, stunting, reduce tillering, necrosis	> 50%	AlJouf, Eastern province (AlAhsa, AlHofuf), AlQassim, Makkah, Riyadh, Baha, Jazan, Tabuk, Madinah, Najran, Asir
10	Potato virus Y (PVY)	*Potyvirus*, *Potyviridae*	potato, tomato, pepper, alfalfa	Aphids *(M. persicae)*, mechanical, sap, and vegetative	yellow mosaic, mottling, stunting, brown/black necrotic streaks on leaves	Up to 80%	Riyadh, Eastern province (AlAhsa, AlHofuf, Abqaiq, AlOyun), Hail, Asir, Najran, Baha, AlQassim, Tabuk, AlJouf, Haradh,
11	Tomato spotted wilt orthotospovirus (TSWV)	*Orthotospovirus*, *Tospoviridae*	tomato, periwinkle (*Catharanthus roseus* L.)	thrips (*T. tabaci*), seeds, and mechanical (grafting)	stunting, bronzing of young leaves, and necrotic streaks on stems	50–90%	Makkah, Jeddah
12	Tomato brown rugose fruit virus (ToBRFV)	*Tobamovirus*, *Virgaviridae*	Tomato, Pepper	mechanical contact (hands, tools, clothing, direct plant contact), seeds, and pollinators like bumblebees	yellowing, mottling, necrosis on leaves, rugose, and deformed fruit.	30–70%	AlQassim, Riyadh, Najran, Makkah, AlJouf, AzZulfi, Tabuk, Asir, Eastern province (AlAhsa, AlHofuf, Abqaiq, Al-Oyun)
13	Cucumber green mottle mosaic virus (CGMMV)	*Tobamovirus, Virgaviridae*	cucurbits, bottle gourd, watermelon	mechanical contact (tools, direct plant contact), seeds, soil,	mosaic, mottling, stunting, and leaf distortion	Up to 70%	Riyadh, AlQassim, Hail,
14	Pepper mild mottle virus (PMMoV)	*Tobamovirus, Virgaviridae*	*C. annuum*	mechanical contact (tools, direct plant contact), seeds, soil,	stunting, leaf puckering, vein clearing, and spots on fruit	Up to 70%	Tabuk and Asir, AlJouf, AlQassim, Eastern Province, Makkah, AlBaha, Madinah, Riyadh

### 3.1 Alfalfa mosaic virus

AMV (genus *Alfamovirus*, family *Bromoviridae*) is endemic to Saudi Arabia’s premier forage crop, alfalfa (*M. sativa*). Since its first detection in 1971 via DAS-ELISA (Abu-Yaman and Abu-Blan, 1973), subsequent detections from Riyadh, AlQassim, Tabuk, and Hail (during 1989–1991), Hail and Wadi AlDawasir (during 2012), Riyadh, AlQassim, Hail, and Haradh (during 2015–2016), and recently from Riyadh (in 2020) (Abdalla et al., 2020), confirm sustained infection cycles. Its persistence is facilitated by extensive host range—exceeding 150 species across 22 families—and perennial reservoirs including *S. nigrum, C. quinoa, and Datura stramonium*.

Alfalfa cultivation in Saudi Arabia, spanning 421,061 hectares in 2018 with a yield of 4,431 metric tons, underpins a market valued at $2.21 billion in 2025, projected to reach $2.85 billion by 2030 (compound annual growth rate [CAGR]; https://www.mordorintelligence.com/industry-reports/saudi-arabia-alfalfa-market. Accessed on March 1, 2025). AMV incur up to 58% yield losses while infecting over 150 plant species across 22 families (Al-Saleh and Amer, 2013; Al-Shahwan et al., 2017; Abdalla et al., 2020). Despite transition from serological assays to RT-PCR, systemic resistance screening, certified seed programs, and weed-reservoir management are still poorly integrated (Al-Saleh et al., 2014c).

### 3.2 Cucumber mosaic virus

CMV (genus *Cucumovirus*, family *Bromoviridae*) shows one of the widest agroecological footprints among Saudi plant viruses. Initially identified in AlQassim and Riyadh (1989–1991) via serological techniques (Salama et al., 1987). Since then, it has consistently been detected across Tabuk, Hail, AlJouf, AlAhsa, and Makkah (1989–2016) (Soliman, 2019), reflecting both its biological adaptability and the diversification in Saudi Arabia’s vegetable production systems (Al-Zahrani et al., 2018).

CMV is widely established across Saudi Arabia due to its efficient spread via aphids, contaminated seeds, sap, and mechanical contacts. Improved diagnostics over time have expanded from DAS-ELISA, electron microscopy, to RT-PCR (Al-Shahwan et al., 1997; Al-Shahwan et al., 2017; Soliman, 2019). In Saudi agricultural system, CMV affects multiple vital crops, including potato, alfalfa, tomato, cucumber, pepper, watermelon, and periwinkle (*C. roseus*)—and numerous wild hosts like *S. oleraceus*, *Vigna unguiculata*, *Hibiscus* spp., *Cucurbita maxima*, and *Chenopodium amaranticolor*. It severely reduces cucumber productivity by disrupting sugar metabolism and chlorophyll levels and incurs up to 50% yield losses (Roossinck, 2001; Shakeel et al., 2016). Its persistence across open-field and protected cultivation systems is aided by aphid transmission, enabling its persistence across diverse agro zones in Saudi Arabia.

### 3.3 Zucchini yellow mosaic virus

ZYMV (genus *Potyvirus*, family *Potyviridae*) is a dominant constraint to cucurbits, including cucumber, melon, watermelon, luffa (*L. acutangula*), pumpkin, squash, bottle gourd, and snake cucumber, in Saudi Arabia. Its continuous detection since the late 1980s (Khan and Alamy, 1986; Al-Shahwan, 1990) from Eastern Province (AlAhsa) in 1987 via serological assays (Salama et al., 1987; Al-Hudaib, 1997), followed by Riyadh, AlQassim, and Hail in 1992 via DAS-ELISA (Al-Shahwan et al., 1995; Al-Hudaib, 1997; Al-Saleh and Al-Shahwan, 1997), and Madinah in 2012 (Saud and Abrahaim, 2012; Al-Saleh et al., 2014d) confirm its widespread and persistent presence nationwide.

ZYMV transmission by aphids and sap poses a major threat to Saudi Arabia’s agriculture, where yields – such as 196,255 tons of cucumber/gherkin and 74,172 tons of squash (*Cucurbita pepo* L.) face declines of up to 80% (Rao and Reddy, 2020; Faostat, 2025), threatening the domestic supply (Lobell et al., 2009). Limited deployment of resistant cultivars and weak seed-health regulation continue to allow chronic yield suppression in high-input cucurbit systems.

### 3.4 Tomato yellow leaf curl virus

TYLCV (genus *Begomovirus*, family *Geminiviridae*) is Saudi Arabia’s most impactful and widely studied plant virus, burdening tomato production since the 1950s (Mazyad et al., 1979). Its diagnostic trajectory—evolving from ELISA (1996) and PCR (2006) to HTS (2024)—reflects its established presence (Al-Shahwan et al., 1997; Al-Shahwan et al., 1997; Ajlan et al., 2007; Sohrab and Daur, 2018; Sattar et al., 2024). TYLCV detections across all major agricultural regions, including Jeddah, Makkah, Tabuk, Jazan, Hadasham, AlQassim, Hail, and AlAhsa (AlHofuf), underscore its extensive geographic spread. It poses a threat to Saudi Arabia’s tomato and chili production, which amounts to 636,650 tons (on 14,597 ha) and 128,925 tons (on 3,458 ha) annually, respectively (Faostat, 2025).

Transmitted by *Bemisia tabaci (B. tabaci)*, TYLCV maintains a broad host range and also infects common bean, wild mint (*M. longifolia*), cucumber, ridge gourds, and chilies, frequently inducing 50–70% yield losses. Despite extensive epidemiological research, molecular diversity, and control strategies (Abdou, 1973; Abul-Hayja et al., 1983; Cook et al., 1984a; Abu-Jawdah, 1986; Al-Besher and Salama, 1991; Salama et al., 1994; Al-Shahwan et al., 1997; Al-Shahwan et al., 2001; Sohrab and Daur, 2018; Sohrab, 2020; Sattar et al., 2024; Iqbal, 2025a), field level management remains inconsistent, necessitating more robust integrated control strategies.

### 3.5 Maize dwarf mosaic virus

Maize dwarf mosaic virus (MDMV; genus *Potyvirus*, family *Potyviridae*) was likely introduced to Saudi Arabia alongside maize (*Zea mays* L.) cultivation and its aphid vector, which transmit the virus non-persistently. It was initially reported on maize during 1987-1988 (El-Meleigi, 1989). MDMV infects key crops, including maize, sorghum, barley, alfalfa, blue panic grass (*P. antidotale*), and rhodes grass (*Chloris gayana* Kunth) – all vital to national food security, livestock production.

MDMV presence from northern (AlJouf), central (AlQassim, Jazan), southern (Jazan, Asir), Eastern (AlHofuf, Riyadh), and western (Makkah, AlBaha, and Madinah) regions highlights widespread distribution and ecological adaptability.MDMV is a persistent threat to maize, covering over 137,745 ha with 922,774 tons annual production (Faostat, 2025). Given the large national footprint of maize and forage crops, MDMV represents a system-level risk rather than an isolated disease problem.

### 3.6 Cucurbit chlorotic yellows virus

Cucurbit chlorotic yellows virus (CCYV; genus *Crinivirus*, family *Closteroviridae*) emerged during the 2014–2015 seasons, first confirmed in cucumber in Riyadh via DAS-ELISA and RT-PCR (Al-Saleh et al., 2015). Subsequent reports from Makkah, Eastern Province, Northern Border, and Najran indicate regional spread. CCYV infects a wide range of cucurbit crops—including cucumber, squash, melon (*Cucumis melo* L.), and watermelon (*Citrullus lanatus*)—as well as weed such as *M. parviflora*, *Lactuca saligna*, and *L. serriola*. In 2014–2015, 61.1% of Riyadh’s cucumber samples tested positive, highlighting its destructive impact (Al-Saleh et al., 2015). CCYV’s efficient transmission combined with widespread presence of *B. tabaci* in Saudi Arabia facilitates its rapid dissemination and control. CCYV causes yellowing and stunting symptoms, leading to reduction in fruit quality, yield and marketability.

### 3.7 Soil-borne cereal mosaic virus

Soil-borne cereal mosaic virus (SBCMV; genus *Furovirus*, family *Virgaviridae*) is a soil-borne virus with long-term persistence mediated by *Polymyxa graminis (P. graminis)*, making detection and management inherently challenging. Globally reported in wheat, rye, and triticale (Budge et al., 2008), but it remains underreported in Saudi Arabia. Nevertheless, recent surveys by the Ministry of Environment, Water, and Agriculture (MEWA) indicate its presence in maize, sorghum (*S. bicolor*), millet (*Panicum miliaceum*), sugarcane (*Saccharum officinarum*), blue panic grass, rhodes grass, barley, and wheat across Hail, AlQassim, and Riyadh (K. Hussain personal communication).

Although cereal, especially wheat, production in Saudi Arabia has declined since the early 2000s due to water scarcity and policy shifts, SBCMV likely arrived historically via imported seeds or soil. This virus causes 20–50% yield losses (Marra et al., 2022), and due to extensive host range and soil persistence, it is a growing threat to food security and sustainable cereal production (Koenig and Huth, 2000).

### 3.8 Watermelon chlorotic stunt virus

WmCSV (genus *Begomovirus*, family *Geminiviridae*) represents a significant threat to Saudi Arabian cucurbits production. Since its first detection in 2014, WmCSV has expanded from Jeddah, AlLith, Tofeel, Asfan, Ghonfada, Jazan, Wadi Baish, and Abu Arish regions (Al-Saleh et al., 2014a), to AlAhsa, Jazan, Tabuk (2013-2014) (Ahmad et al., 2018), AlHofuf, AlLith, AlQateef, and Dammam (2022) (Ahmad et al., 2018; Alhudaib et al., 2018; Rezk et al., 2019; Hussain et al., 2022; Amer et al., 2024; Sattar et al., 2024). The long-standing history and extensive distribution—from coastal regions of Jeddah and Jazan to the inland hubs of AlAhsa and Tabuk—highlights the WmCSV’s adaptability to diverse agroecological zones (Iqbal, 2025a).

WmCSV seriously endangers watermelon, cultivated over 25,198 hectares with an annual yield of 612,680 tons (Faostat, 2025), also infects melon, squash, and cucumber (Rezk et al., 2019). Transmitted persistently by *B. tabaci*, WmCSV disrupts physiological development, causing up to 90% yield losses of watermelon. As part of a regional *begomovirus* complex, WmCSV often coinfects with TYLCV, intensifying crop damage.

### 3.9 Barley mild mosaic virus

Barley mild mosaic virus (BaMMV; genus *Bymovirus*, family *Potyviridae*) is another emerging soil-borne pathogen of cereal and forage crops. It persist across Saudi Arabia’s diverse agro-ecological zones, including AlJouf, Eastern Province, AlQassim, Makkah, Riyadh, AlBaha, Jazan, Tabuk, Madinah, Najran, and Asir (Khalid et al., 2024). Its extensive host range—encompassing strategic cereals (wheat, barley, maize, and sorghum) and key forage species (*P. antidotale* and *Chloris gayana*)—poses a systemic threat to both food security and livestock supply chains.

BaMMV transmission by *P. graminis* and the combination of soil persistence, broad host range, and limited awareness underscores BaMMV as a system-level threat requiring greater inclusion in national surveillance and seed-health frameworks.

### 3.10 Potato virus Y

PVY (genus *Potyvirus*, family *Potyviridae*) represents a substantial economic threat to Saudi Arabia’s potato production, which spans 21,287 hectares and yields 610,390 tons annually (Faostat, 2025). Initially reported in the Kingdom in 1984 (Cook et al., 1984b), subsequent surveys (1989-2019) using ELISA, electron microscopy, and RT-PCR (Al-Saikhan et al., 2014; Chikh-Ali et al., 2016) have confirmed its endemic status across diverse agro-ecological zones—from the temperate highlands of Tabuk and Hail (Al-Besher and Salama, 1991) to the arid lowlands of Riyadh (2010), Haradh, Hail, Jeddah (2013), AlJouf (2014), to AlQassim, AlAhsa, Hail, and Wadi AlDawasir (2019) (Sabir, 2012; Al-Saikhan et al., 2014; Chikh-Ali et al., 2016; Rezk et al., 2021) via ELISA (Al-Shahwan et al., 1997).

While potato is primary host, PVY also infects other solanaceous crops (tomato, pepper, and tobacco), though these hosts remain underreported locally. PVY exhibits high pathogenicity; aggressive strains like PVY^NTN^ and PVY^N-Wilga^ cause potato tuber necrotic ringspot disease (PTNRD), responsible for global yield losses of up to 80% (Karasev and Gray, 2013; Chikh-Ali et al., 2016). PVY spreads non-persistently via aphid vectors, notably *Myzus persicae (M. persicae)*, whose perennial activity is sustained by the Kingdom’s warm climate. PVY broad geographic range demonstrates remarkable ecological adaptability and demands continuous surveillance and resistance deployment.

### 3.11 Tomato spotted wilt orthotospovirus

TSWV (genus *Orthotospovirus*, family *Tospoviridae*), a destructive pathogen, was first detected in AlQassim and Riyadh (1991) using serological methods (Salama et al., 1994; Al-Shahwan et al., 1998). Later, it was identified in Hail, Tabuk, AlHofuf, Najran, AlUyaynah, and Taif via ELISA and RT-PCR (Al-Saleh et al., 2014b; El-Shazly et al., 2016). TSWV infects a wide range of crops, including eggplant, potato, lettuce, tomato, and purple goosefoot (*C. ambrosioides*). It incurs severe yield losses (50–90%) by its persistent transmission via by thrips, notably *Frankliniella occidentalis* and *Thrips tabaci*, complicating its management due to their broad host range (Whitfield et al., 2005).

Economically, TSWV threatens Saudi Arabia’s vegetable sector, and its persistence is reinforced by year-round thrips populations. While basil essential oil, *Plantago major* extract, and lupenone show promising management (up to 64%) but integrated field-level management strategies remain underdeveloped (El-Shazly et al., 2016; Elgorban et al., 2023).

### 3.12 Tomato brown rugose fruit virus

ToBRFV (genus *Tobamovirus*, family *Virgaviridae*) has recently emerged as a serious plant pathogen in Saudi Arabia. First identified in Riyadh (2021–2022) infecting tomato plants symptoms (Sabra et al., 2021), the virus introduced in Saudi Arabia via international seed trade or mechanical transmission. Currently confirmed from Riyadh, AlKharj, AzZulfi, AlQassim, Asir, Makkah, and AlHareeq, primarily infecting tomato and pepper crops, could spread nationwide due to its seedborne and highly contagious nature (Sabra et al., 2022).

ToBRFV poses a formidable challenges to Saudi Arabia’s tomato and pepper industries–sectors vital to national food security. By rendering produce unmarketable, ToBRFV accounts for global yield losses of 30–70% (Sabra et al., 2022; Khalid et al., 2024), severely disrupting market supply and local agricultural stability.

### 3.13 Cucumber green mottle mosaic virus and pepper mild mottle virus

CGMMV and PMMoV, both members of the genus *Tobamovirus* (family *Virgaviridae*), pose formidable challenge to Saudi Arabian cucurbit and pepper crops. CGMMV was first reported in Dirab (Riyadh) in 1992 and subsequently confirmed in AlQassim and Hail (1992-1993), with recurrent detections in Riyadh and Hail in 2012 via and RT-PCR (Al-Shahwan et al., 1997; Amer, 2015). Its high transmissibility—via contaminated seeds, soil, and mechanical contact makes control difficult. As watermelon and bottle gourd are economically vital, CGMMV’s impact undermines sustainable agriculture goals.

PMMoV exhibits high widespread distribution in nine regions, with high incidence in Tabuk, Asir, AlJouf and AlQassim, moderate prevalence in the Eastern Province, Makkah, and AlBaha, and low rates in Madinah and Riyadh (Alwabli et al., 2017). PMMoV recent detections from AlQassim and AlBaha, via ELISA and RT-PCR, confirms its ongoing impact (Khalid et al., 2024).

The epidemiological synergy of these two viruses—characterized by seed-borne transmission, mechanical spread, high environmental stability, and long-term persistence—exposes systemic vulnerabilities in resistance deployment and sanitation protocols. Despite the economic importance of watermelon, bottle gourd, and pepper, these pathogens remain insufficiently addressed within current management frameworks, posing a critical risk to intensive and protected cultivation systems.

## 4 Spatial distribution and host associations of economically important plant viruses

**Figure 4:**
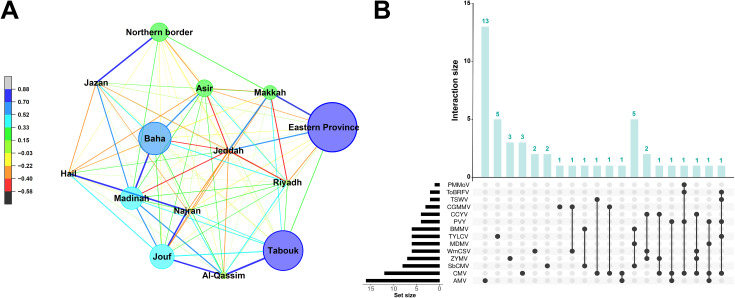
**(A)** Network visualization of plant virus distribution across 13 major agricultural regions of Saudi Arabia. Network analysis was performed in OriginPro v2024 using Pearson correlation coefficients calculated across regional virus prevalence – retrieved from MEWA website. Node size indicates regional viral incidence, while edge thickness and color represent the strength and direction of correlations (r = −0.58 to 0.88). Blue edges denote strong positive associations, reflecting shared viral populations, whereas red to black edges indicate weak or negative correlations. The color gradient (left) corresponds to Pearson correlation values among regional virus prevalence patterns. **(B)** Prevalence and co-occurrence of major plant viruses from different hosts in Saudi Arabia. UpSet analysis was conducted using ChiPlot (https://chiplot.online/) using the default parameters/setting on the website. Horizontal bars indicate total detections per virus, and vertical bars represent co-occurrence.

A network analysis of economical important plant viruses (section 3) occurrence across 13 major agricultural regions of Saudi Arabia revealed distinct geographic clustering patterns, non-random spatial structure occurrence, and correlation strengths (**Figure 4A**). The Eastern Province, Tabuk, and AlBaha emerged as central hubs, showing high viral richness, strong node connectivity, and strong positive correlations with multiple regions, particularly Riyadh, AlQassim, and Madinah. These regions likely serve as major centers for viral spread due to intensive crop cultivation, greenhouse production, and interregional seed or plant movement. In contrast, Northern Border and Makkah displayed weaker network integration, indicating limited viral diversity or lower surveillance activity. The predominance of blue colored edges reflects significant co-occurrence of viral species across neighboring regions, implying shared environmental drivers or vector-mediated transmission pathways. The clustering pattern underscores the influence of climatic similarity, agricultural intensification, and vector ecology on virus distribution dynamics. Some regions, like Northern border and Makkah, exhibited weaker integration, which indicated either lower viral pressure or underrepresentation in surveillance rather than true epidemiological isolation. Collectively, this network demonstrates the interconnected nature of plant virus epidemiology in Saudi Arabia.

The UpSet plot (**Figure 4B**) complements and illustrates these spatial patterns, frequency, and intersection of economically important plant viruses detected from various hosts in Saudi Arabia. Among the identified viruses, AMV and CMV were the most detected viruses in multiple host species, reflecting their wide host adaptability and high incidence. Frequent intersections involving SBCMV, ZYMV, WmCSV, MDMV, TYLCV, and BaMMV indicate that key crops are exposed and vulnerable to overlapping viral pressures rather than single virus outbreak. Such co-occurrences are epidemiologically important, as mixed infections can intensify disease severity through synergism, compromise resistance durability by eroding R-genes, and complicate the management strategies. Addressing this requires a paradigm shift toward multi-virus surveillance and breeding programs resilient to concurrent stresses.

## 5 Advances in detection and management

**Figure 5:**
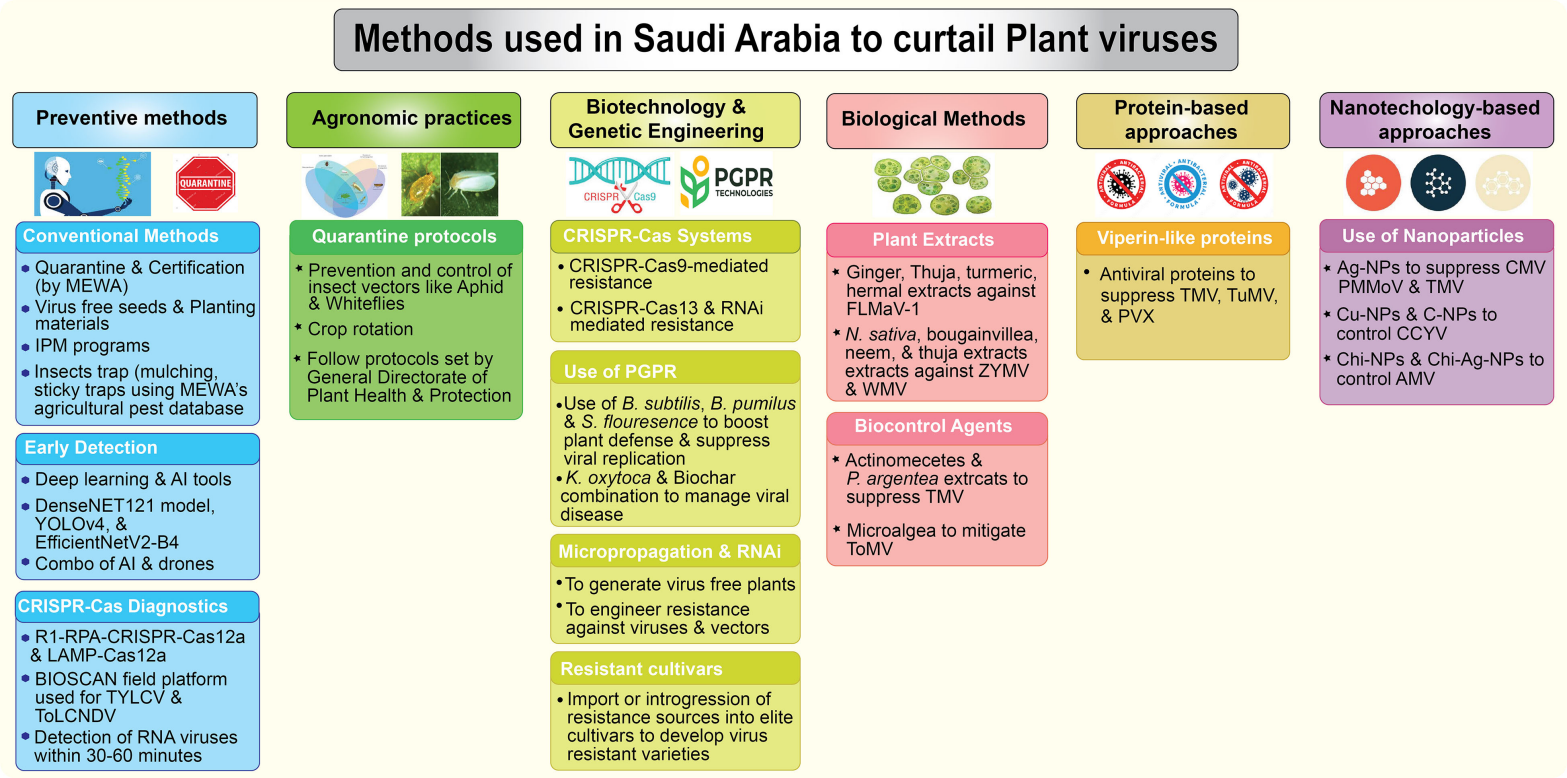
Overview of methods used in Saudi Arabia to curtail plant viruses. Six key approaches have been employed in Saudi Arabia to manage plant viral diseases.

Detection and management of plant viruses in Saudi Arabia have advanced significantly. This section reviews key strategies to manage plant viral diseases (**Figure 5**) and critically evaluates their current readiness, scalability, and regulatory feasibility within the Saudi Arabian context (**Supplementary Table S2**).

### 5.1 Preventive methods

Preventive strategies are essential for limiting virus introduction and spread in Saudi Arabia’s arid agroecosystems, emphasizing early detection and regulatory measures.

#### 5.1.1 Conventional approaches

Saudi Arabia implements rigorous quarantine and seed certification protocols through MEWA to ensure virus free imports. Integrated pest management (IPM) programs support these efforts through vector controls—such as the use of colored insect traps—and regular farmers training, bolstered by a national agricultural pest database for informed decision-making in virus-prone crops.

#### 5.1.2 Early detection using AI and deep learning

Artificial intelligence and deep learning have been employed for virus detection in Saudi Arabia. Alzahrani and Alsaade (2023) showcased the DenseNet121 model’s ability to identify tomato mosaic virus (ToMV) and TYLCV by analyzing leaf symptoms like mosaic patterns and curling (Alzahrani and Alsaade, 2023). While YOLOv4 tool (Aldakheel et al., 2024) and customized AI tool, EfficientNetV2B4, in conjunction with the Plant Village Kaggle dataset, achieved a remarkable 99.93% precision and accuracy in identifying a range of plant diseases (Albattah et al., 2022). AI and drones reduce chemical use by up to 40% and boost yields by 20%, enabling real-time monitoring in regions like Jeddah, Riyadh, and AlAhsa (Puspitasari et al., 2024).

#### 5.1.3 CRISPR–Cas based nucleic acid diagnostics

CRISPR technology revolutionizes rapid, field-deployable plant virus diagnostics in Saudi Arabia. Aman et al. (2020) introduced an RT-RPA-CRISPR-Cas12a assay detecting diverse RNA viruses (potexviruses, potyviruses, tobamoviruses) at a single temperature in under 30 minutes, simplifying in-field use (Aman et al., 2020a; Aman et al., 2020b). Extending this, Mahas et al. (2021) developed a LAMP-coupled CRISPR-Cas12a assay for early TYLCV and tomato leaf curl New Delhi virus detection in tomatoes, providing visual readouts in one hour via cost effective fluorescence (Mahas et al., 2021). The BIOSCAN platform demonstrates robust viral diagnostic capability, enabling accurate detection of DNA viruses such as TYLCV and RNA viruses including TMV and PVY across different plant hosts (Sánchez et al., 2022). These sensitive assays enable timely pathogen identification, supporting quarantine and management in virus-prone regions such as Eastern Province, Tabuk, Riyadh, and AlBaha to protect tomato production.

### 5.2 Agronomic practices

Agronomic interventions form the frontline defense, disrupting virus lifecycles through cultural and management tactics tailored to Saudi Arabia’s intensive farming systems.

#### 5.2.1 Quarantine protocols

To mitigate viral outbreaks, the General Directorate of Plant Health and Protection (2019) enforces stringent quarantine measures targeting insect vectors—primarily aphids and whiteflies—responsible for spreading major viruses such as TYLCV, WmCSV, and AMV. These protocols operate at national borders, inspection stations, and key agricultural hubs including AlQassim, Riyadh, the Eastern Province, Tabuk, and Hail, effectively curbing viral introductions and regional dissemination.

#### 5.2.2 Crop rotation and vector management

Crop rotation and vector management remain cornerstone practices for controlling AMV, CMV, TYLCV, and TSWV. By rotating high-value crops like tomato and cucumber with nonhost species, farmers reduce pest reservoirs and vector populations (aphids, thrips, and whiteflies), particularly in Riyadh, AlQassim, and the Eastern Province. These integrated measures help minimize yield losses and sustain vegetable production systems.

### 5.3 Biotechnology and genetic engineering approaches

Biotechnological and genetic engineering are transforming plant virus management, offering precision tools to engineer resistance, suppress vectors, and reduce chemical reliance. Most of the biotechnological interventions have been employed successfully but only under controlled glasshouse conditions. While global tools like HTS, CRISPR diagnostics, and AI surveillance dominate internationally, their Saudi deployment faces cost, regulatory, and capacity constraints. Bridging this gap requires phased integration via pilot programs, capacity building, and regulatory adaptation to align technology with operational readiness.

#### 5.3.1 CRISPR-Cas systems for virus resistance

CRISPR-Cas-based systems have revolutionized crop protection through precise, adaptable genome editing. At King Abdullah University of Science and Technology (KAUST), Ali et al., (2015, Ali et al., 2016) pioneered CRISPR-Cas9mediated resistance against geminiviruses, followed by Tashkandi et al. (2018) who engineered TYLCV resistance in *N. benthamiana* (Ali et al., 2015b; Ali et al., 2016; Tashkandi et al., 2018). Aman et al., 2018, Aman et al., 2020) expanded these advances by combining CRISPR-Cas13a with RNAi to target turnip mosaic virus (TuMV) and later developing rapid RT-RPA-Cas12a assays for plant RNA virus detection (Aman et al., 2018, Aman et al., 2020b). Mahas et al. (2019) further introduced virus-mediated genome editing (Mahas et al., 2019). Collectively, these lab-scale studies position CRISPR-Cas systems as pivotal in building viral resistance and advancing Saudi Arabia’s biotechnology-driven agriculture.

Notably, CRIPSR-mediated engineered resistance approaches remained at experimental stages and have not progressed to field deployment. This limitation highlights a significant gap between virological surveillance capacity and resistance breeding infrastructure in the Kingdom, representing an important priority under Vision 2030 agricultural sustainability initiatives.

#### 5.3.2 Micropropagation and RNAi

Micropropagation and RNA-based technologies are gaining momentum in Saudi Arabia as innovative tools for managing plant viral diseases and their insect vectors. Micropropagation and RNAi technologies provide effective virus elimination and vector suppression strategies. In Makkah, Alzahrani and Alsaade (2023) eliminated FMV from *F. carica* via apical meristem culture (Al-Zahrani et al., 2018). In Riyadh and AlQassim (Faisal et al., 2021), developed aphid-resistant transgenic tomatoes using RNAi to silence *Ace1* in *M. persicae*, significantly reducing vector populations (Faisal et al., 2021). These molecular tools support clean plant production and vector control in key horticultural systems, but remain limited to lab-scale.

#### 5.3.3 Virus-resistant germplasm

The development of virus-resistant germplasm is a cornerstone for the sustainable management of plant viral diseases within the unique agro-ecological landscape of Saudi Arabia. Despite its importance, domestic efforts in this sector remain nascent and are characterized by several critical gaps. To date, the prevailing research trend in the Kingdom has been descriptive rather than developmental; the majority of published studies focus on screening existing exotic or local varieties to assess their performance under regional viral pressure. While these screening efforts provide essential baseline data on susceptibility, there is a profound lack of active breeding programs dedicated to the *de novo* development of resistant lines.

Naser et al. (2020) evaluated imported alfalfa cultivars in Tabuk and Hail, identifying strong resistance to alfalfa enation virus (AEV) that significantly reduced yield losses (Naser et al., 2020). Screening of seven indigenous watermelon cultivars against watermelon mosaic virus (WMV-SA) isolate deciphered that all the cultivars are susceptible and showed mild-to-severe symptoms with a reduction in biomass and growth (Al-Shahwan et al., 2018). For tomato, Al-Saikhan et al. (2020) demonstrated that Ty-2 gene confers resistance to TYLCV-Has and TYLCV-IL strains but not TYLCV-Pep, with heterozygous (Ty-2/ty2) and homozygous (Ty-2/Ty-2) genotypes showing equal protection (Al-Saikhan et al., 2020). Conversely, Sabra et al. (2022) found no resistance to ToBRFV among 13 tested tomato cultivars, with disease severity indices ranging from 52-96% (Sabra et al., 2022). In a recent screening of 13 commercial Saudi tomato cultivars, Amer et al. (2025) demonstrated that all tested varieties were susceptible to both TYLCV and Tomato leaf curl Sudan virus (TLCSDV), with the infected plants exhibiting varying degrees of symptom severity. These findings highlight both the potential of resistance breeding and critical gaps—particularly the absence of comprehensive germplasm screening programs, limited molecular marker development, and lack of locally adapted resistant varieties for major threats like WmCSV, ZYMV, and PVY.

### 5.4 Biological control methods

Biological control methods offer sustainable alternatives to synthetic pesticides, harnessing plant-derived extracts and microbial agents adapted to Saudi Arabia’s arid ecosystems.

#### 5.4.1 Plant extracts

Plantderived compounds, especially from medicinal and aromatic plants have demonstrated notable antiviral activity against a range of plant viruses. Extracts from Thuja (*Thuja occidentalis*), ginger (*Zingiber officinale*), Harmal (*Peganum harmala*), and turmeric (*Curcuma longa*) suppressed fig leaf mottle-associated virus 1 (FLMaV1) in fig orchards of West Makkah (Aldhebiani et al., 2017). Similarly, extracts from *Nigella sativa*, bougainvillea, neem (*Azadirachta indica*), and Thuja effectively reduced ZYMV incidence in cucurbits in Makkah (Elbeshehy, 2017). *Thuja orientalis* extracts also mitigated WMV and lowered vector abundance in Riyadh (Elbeshehy et al., 2015a), demonstrating the promise of bioactive botanicals in integrated virus management.

#### 5.4.2 Biocontrol agents

Biocontrol agents are another cornerstone of biological disease management. Abdelkhalek et al. (2021) employed *Actinomycetes* and *Paronychia argentea* extracts to suppress TMV in tomato fields in the AlQassim region (Abdelkhalek et al., 2021). Likewise, Elsharkawy et al. (2022) demonstrated the efficacy of microalgae in mitigating ToMV in Jeddah (Elsharkawy et al., 2022). These biological agents reduce pesticide reliance, complement resistant cultivars, and foster ecological balance within Saudi agroecosystems.

PGPR offer a sustainable, ecofriendly approach to manage viral diseases in Saudi Arabia’s arid environment. These beneficial microbes enhance plant resilience not only by improving growth and nutrient uptake but also by activating broad-spectrum defense mechanisms against viral pathogens. PGPRs, including *Pseudomonas fluorescens* and *Bacillus subtilis*, strengthen antiviral defense via systemic resistance pathways (SAR and ISR), activating genes such as NPR1, COI1, and PR1a (Montasser et al., 2006; Sofla et al., 2023). They also produce antibiotics, siderophores, and lytic enzymes that suppress viral replication and insect vectors (Sudhakar et al., 2011). Field trials across Riyadh, Jazan, AlAhsa, and Makkah confirmed reduced CMV, ToMV, and WMV severity and improved yields (Elbeshehy et al., 2015b; Sofla et al., 2023). PGPR-based bioformulations thus offer ecofriendly protection aligned with sustainable farming initiatives.

### 5.5 Protein-based approaches for virus control

Protein-based antiviral strategies represent a new frontier for environmentally safe virus control. Notably, Kamel et al. (2024) investigated the antiviral potential of Viperin-like proteins to suppress RNA viruses, including TMV, TuMV, and PVX, in potato and tomato crops. Their study conducted in AlQassim and Riyadh demonstrated varying levels of viral interference through transient and stable overexpression in *N. benthamiana* (Kamel et al., 2024). Such protein-based defenses can offer effective, low-impact alternatives to chemical control, advancing sustainable plant protection in arid environments.

### 5.6 Nanotechnology-based approaches

Nanotechnology provides precise, sustainable tools for antiviral defense in Saudi Arabia’s arid agroecosystems. Silver nanoparticles (AgNPs) disrupted CMV replication in cucumber (AlAhsa) (Elbeshehy et al., 2021), while *Punica granatum*-derived AgNPs effectively controlled TMV in tomato (Riyadh) by targeting viral coat proteins (Al-Askar et al., 2023). Copper and carbon-based nanoparticles suppressed CCYV in cucurbits (Jazan), reducing pesticide reliance (Al-Zaban et al., 2022). Chitosan and chitosan–silver nanocomposites further decreased AMV infections (El-Ganainy et al., 2023), and chemically synthesized AgNPs mitigated PMMoV in pepper seedlings (Elbeshehy et al., 2023). These nanoparticles, with high surface area-to-volume ratios, bind efficiently to viral particles or vectors such as whiteflies and aphids, prevalent in Saudi Arabia’s warm environments. Field evaluations across Riyadh, Jazan, AlAhsa, AlQassim, and Tabuk confirm their broad adaptability and efficacy. Overall, nanotechnology offers a viable, ecofriendly alternative to conventional pesticides, enhancing crop protection, sustainability and agriculture resilience.

## 6 Research capacity and disciplinary evolution of plant virology

Plant virology in Saudi Arabia has undergone remarkable development over the past two decades, transitioning from foundational diagnostics to an integrated, molecular- and genomics-driven discipline. This progress reflects growing institutional capacity, a highly trained research community, and increasing integration with global virology networks.

Historically, plant virus research in Saudi Arabia has transitioned from traditional methods to the integration of molecular and biotechnological approaches. Initially, virus identification relied on visual symptom assessment and serological assays. Early research in the 1970s documented key viruses such as TYLCV in tomato (Mazyad et al., 1979), AMV in alfalfa (1971), PVX (1989–1991) (Abu-Yaman and Abu-Blan, 1973), CMV and ZYMV (Khan and Alamy, 1986; Al-Shahwan and Abdalla, 1992; Al-Saleh and Al-Shahwan, 1997) using field diagnostics, agar diffusion tests, and DAS-ELISA. These pioneering efforts laid the foundation for modern molecular virology.

The advent of PCR and RT-PCR in the 1990s and its adoption in the 2000s marked a critical methodological shift. This molecular transition supported detection of PVY in Riyadh and Jeddah (Al-Shahwan et al., 1997), and RNA viruses such as GarV−A, GarV−D, and FMV across Riyadh, AlBaha, and Makkah (Aldhebiani et al., 2015; Amer et al., 2024; Amir et al., 2024), enhancing understanding of virus genetics, epidemiology, and vector transmission in arid conditions.

In the past decade, the adoption of HTS, rolling circle amplification (RCA), and CRISPR-Cas technologies has further redefined plant virology in Saudi Arabia. HTS combined RCA with has revealed new strains of TYLCV, CLCuGeV, and WmCSV in Jeddah and AlAhsa (Idris et al., 2014; Alhudaib et al., 2022; Sattar et al., 2024). CRISPR–Cas based systems now complement HTS, enabling rapid, and precise detection aligned with technological innovation goals (Aman et al., 2020b; Mahas et al., 2021).

Beyond methodological advances, Saudi plant virology increasingly focuses on applied outcomes and interdisciplinary collaboration. Research on PepMoV and ToBRFV in peppers and tomatoes has laid the groundwork for developing resistant cultivars and IPM strategies. Strengthening collaborative research among virologists, entomologists, and agronomists at KSU, KFU, and KAUST—with strategic support from MEWA—is now essential to advance insect vector control and enhance crop resilience under Saudi Arabia’s challenging agroclimatic conditions. The field’s adaptability is evident in the dynamic spatial shift of viral prevalence—from CMV and ZYMV dominating the 1980s to the recent emergence of CaCV and ToBRFV (Amer et al., 2024; Amir et al., 2024; Khalid et al., 2024).

Institutionally, Saudi Arabia’s strength lies in its centralized molecular infrastructure and coordinated research efforts. Leading universities and research centers—such as King Saud University (KSU), King Abdullah University of Science and Technology (KAUST), and King Faisal University (KFU)— account for the majority of national plant virology output since 2015. These institutions operate modern laboratories for viral detection, genome sequencing, and vector management, supported by MEWA through its surveillance and extension programs. KSU’s Department of Botany and Microbiology has elucidated the molecular diversity of viruses infecting tomato, garlic, and alfalfa, including ToBRFV, allexivirus, and GarV−D (Aldakheel et al., 2024; Amer et al., 2024; Amir et al., 2024). At KAUST, pioneering work on CRISPR–Cas systems has positioned Saudi Arabia at the forefront of developing gene editing–based antiviral technologies (Ali et al., 2015b; Aman et al., 2020b). The adoption of HTS by KFU and MEWA laboratories has enabled simultaneous detection of multiple viral pathogens and the identification of novel begomoviruses and mixed infections across major crop systems (Idris et al., 2014; Alhudaib et al., 2022; Sattar et al., 2024).

Beyond institutional capacity, Saudi researchers have demonstrated growing international visibility—serving as editors and peer reviewers for leading journals and publishing in platforms such as Viruses, Frontiers in Virology, and Plant Pathology. The emergence of inter-institutional collaborations and data-sharing initiatives now provides a foundation for developing a national plant virus database and for strengthening biosecurity frameworks. Collectively, these developments highlight a clear transition from fragmented, descriptive diagnostics to data-driven, innovation-oriented virology. The integration of molecular biology, bioinformatics, and biotechnology within coordinated national programs positions Saudi Arabia to lead regional efforts in viral surveillance, early detection, sustainable crop protection, food security, and agricultural resilience.

## 7 Plant viruses, sustainable agricultural, and Vision 2030

As Saudi Arabia advances toward the objectives of Vision 2030, emerging plant viruses like AMV, TYLCV, WmCSV, and ToBRFV incur 30–50% yield losses in key Saudi crops, directly undermining agricultural self-sufficiency (Alhudaib et al., 2022). Agriculture is Saudi Arabia operates under inherently fragile conditions, characterized by aridity, salinity, water scarcity, and extreme temperatures. This scenario is aggravated with the viral plant diseases that exert disproportionate impacts on productivity, crop quality, and input efficiency, directly influencing food security and economic resilience.

Globally, plant viruses cause over $30 billion in annual crop losses (Savary et al., 2019; Tatineni and Hein, 2023). In Saudi Arabia, limited epidemiological data obscure the full losses assessment, yet rising vector-borne and seed-transmitted infections indicate growing vulnerability. Despite over $24 billion invested in agricultural development (Ministry of Economy and Planning, 2023), reliance on pesticide-intensive management misaligns with core Vision 2030 sustainability goals – including a 40% reduction in water use and tripling organic cultivation area (Saudi Green Initiative, 2021). These impacts jeopardize food security objectives constraining local production and increasing dependency on imported produce.

Under Vision 2030, improving agricultural efficiency and reducing reliance on food imports, so effective management of plant viruses is not peripheral but a core component of sustainable agriculture. To address challenges, a transition to technology driven, climate smart agriculture is imperative. In this context, early warning systems based on molecular diagnostics, HTS, and emerging AI-assisted surveillance offer practical mechanisms for timely identification of outbreaks, discrimination of mixed infections, and spatially targeted intervention. Such approaches enable site-specific disease management, reduce unnecessary chemical inputs, and support water-efficient production systems. To fully realize these benefits, plant virology research in Saudi Arabia must move beyond fragmented reporting toward coordinated, impact-driven implementation, including the targeted deployment of advanced diagnostics in major production zones, integration of virus surveillance with seed certification and quarantine frameworks, and explicit incorporation of virus pressure into breeding programs for locally adapted cultivars. Strengthened coordination among research institutions, regulatory agencies, and extension services will be essential to translate virological data into actionable management strategies, thereby aligning plant virus research with national priorities in agricultural sustainability, biosecurity, and innovation-led economic diversification.

Beyond surveillance, long-term sustainability requires strengthening genetic resistance within locally adopted crops. At present, Saudi agriculture remains heavily dependent on imported resistant cultivars, creating vulnerabilities in biosecurity and supply chains. Strategic investment in resistance breeding—particularly through CRISPR-assisted approaches—can reduce this dependence, enhance local cultivar development, and support food security objectives. However, the deployment of such technologies must be selective, focusing on high-impact virus–crop systems, and aligned with robust regulatory and biosafety frameworks (Al-Gaadi et al., 2016). IPM approaches now prioritize biological control, using predatory insects and microbial antagonists to suppress aphid and whitefly populations—the primary vectors of CMV and TYLCV—while minimizing chemical inputs.

Plant virus sustainability is also closely linked to climate change. Rising temperatures, altered precipitation patterns, and expanding protected cultivation are likely to increase vector abundance and prolong transmission seasons. Integrating vector ecology, climate data, and digital surveillance into national risk models (like Reef program) will improve predictive capacity and resilience, allowing agriculture to adapt proactively rather than reactively (Al-Zahrani et al., 2016; Alotaibi et al., 2024).

Plant virus management is central to agricultural sustainability under Vision 2030. By prioritizing evidence-based surveillance, targeted resistance breeding, and vector-informed, climate-aware research, Saudi Arabia can shift from reactive, pesticide reliant practices to predictive, data-driven, ecologically balanced viral management. Aligning plant virology with national sustainability agendas will strengthen food security, and long-term agrifood resilience.

## 8 Future directions for plant virology in Saudi Arabia

**Figure 6:**
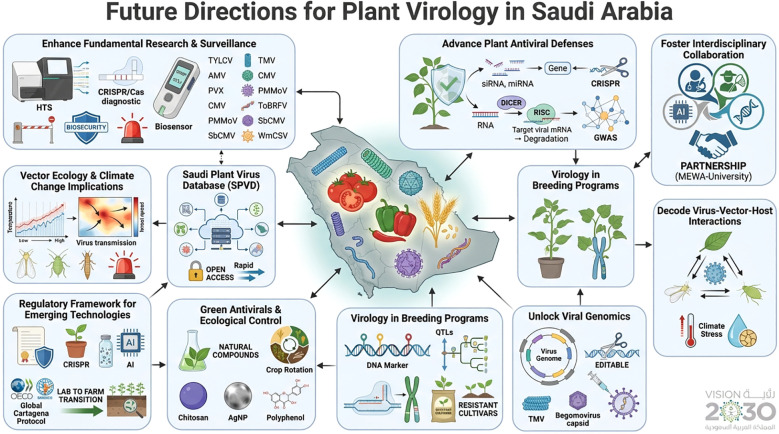
Future research directions for plant virology in Saudi Arabia for the sustainability of agricultural productivity.

Saudi Arabia stands at the threshold of transforming plant virology into a globally competitive discipline that integrates molecular, ecological, and computational approaches to safeguard crop production. Over the next decade, targeted investments, collaborative frameworks, and capacity building initiatives can align research with national priorities for agricultural sustainability, food security, and biosecurity (**Figure 6**).

### 8.1 Enhance fundamental research and surveillance

Future progress hinges on robust, field ready diagnostics and surveillance networks. Advanced tools—HTS, CRISPR-based diagnostics, and portable biosensors—should replace symptom-based detection to provide rapid, affordable, and real-time monitoring. Strengthening quarantine systems and early warning networks under MEWA will help prevent cross-border viral incursions. Early detection and containment of viral outbreaks to protect national crop production and trade biosecurity.

Comprehensive genomic studies of major and emerging viruses—including TYLCV, AMV, PVX, CMV, PMMoV, ToBRFV, SBCMV, and WmCSV—are essential to decipher their replication, host range, and adaptation to the Kingdom’s hot, vector rich environments. Such knowledge will enable gene targeted resistance strategies, supporting evidence-based biosecurity and global leadership in functional virology.

### 8.2 Advance plant antiviral defense mechanisms

Understanding and enhancing innate plant immunity represents a cornerstone of sustainable virus management. Future work should dissect RNA silencing pathways, resistance (R) gene–viral effector interactions, and non-host resistance mechanisms in economically important crops. Building on classical AMV serological studies (Abu-Yaman and Abu-Blan, 1973), genome wide association studies (GWAS) and CRISPR-Cas editing can now identify or engineer broad spectrum resistance genes. Given Saudi Arabia’s dependence on imported seed, strengthening nonhost resistance signaling is vital to block exotic virus establishment and secure domestic seed systems.

### 8.3 Foster interdisciplinary collaborations for novel antiviral solutions

Future virology in Saudi Arabia must be integrative—linking virology, entomology, bioinformatics, nanotechnology, and artificial intelligence (AI). Bioinformatics-driven analyses, such as Iqbal (2025) on TYLCV diversity (Iqbal, 2025b; Iqbal, 2025a), exemplify this trend. AI-enabled outbreak modeling, nanomaterial-based antivirals, and synthetic gene circuits exemplify next-generation strategies. Establishing joint MEWA–university research grants and graduate training networks will ensure talent development and translational outcomes.

### 8.4 Decode virus-vector-host interactions

Understanding the signaling dynamics among viruses, vectors (whiteflies, aphids), and host plants under heat and drought stress is critical. Climate-induced vector expansion necessitates predictive epidemiological models to guide precision IPM strategies. Integrating ecological data with vector genomics will optimize control strategies suited to arid ecosystems.

### 8.5 Unlocking viral genomics

Reverse genetics, CRISPR-mediated genome editing, and virus-derived vectors are transformative tools for functional virology. Reverse genetics systems for TYLCV, CMV, and WmCSV can elucidate viral replication and host interactions. CRISPR-Cas-based editing of susceptibility genes—already successful in engineering TYLCV resistant tomatoes and PepMoV resistant peppers (Ali et al., 2015a; Aman et al., 2018; Tashkandi et al., 2018; Mahas et al., 2019)—should be expanded to other major crops.

Additionally, plant virus based vectors derived from TMV and begomoviruses (Iqbal et al., 2021) provide rapid systems for transient gene expression, RNA interference, and protein production. Addressing vector instability and nonheritable expression will enhance their reliability for molecular farming while ensuring ecological safety.

### 8.6 Translating virology into breeding programs

Integrating virology with breeding through mapping of resistance genes and quantitative trait loci (QTLs) can accelerate development of elite, virus resistant cultivars. CRISPR-Cas technology offers a route to remove susceptibility alleles and introduce durable, region-specific resistance. Integrating these insights into breeding pipelines through QTL mapping and genome editing will generate resistant cultivars combining viral and abiotic stress tolerance.

### 8.7 Develop green antivirals, ecological control, and genomic database

Future virus management should emphasize eco-safe antivirals, natural plant derived compounds, and biological antagonists over chemical pesticides. Research on chitosan, AgNPs, and polyphenol based agents (Aldhebiani et al., 2017; Al-Askar et al., 2023) must continue alongside ecological interventions such as crop rotation and microbial biocontrol.

### 8.8 Establishment of Saudi Plant Virus Database

A centralized Saudi Plant Virus Database should consolidate genomic, epidemiological, and diagnostic data across regions and host species. This open access resource—developed under MEWA coordination—would enable rapid data sharing, viral evolution modeling, and collaborative research. Coupled with national surveillance funding, the SPVD will serve as a cornerstone of Saudi biosecurity infrastructure. Furthermore, it would strengthen national biosecurity and global data visibility, establishing Saudi Arabia as a model in plant virology.

### 8.9 Vector ecology and climate change implications

Future Saudi plant virology must shift from detection to predictive frameworks integrating vector ecology and climate dynamics. Knowledge gaps exist regarding how rising temperatures and alter precipitation affect whitefly, aphid, and thrip populations—potentially extending transmission windows and intensifying virus pressure. Climate-driven shifts may facilitate new virus-vector complexes in arid systems. Integrating longitudinal vector monitoring with climatic and virus surveillance data will enable early warning systems and climate-resilient crop protection strategies aligned with Vision 2030.

### 8.10 Regulatory framework development for emerging technologies

Saudi Arabia’s biosafety regulations inadequately address CRISPR-edited crops, nanotechnology antivirals, and AI diagnostics, hindering virus management modernization. Future frameworks must establish risk-based assessments aligned with international standards (Cartagena Protocol, OECD guidelines). Priorities include expedited approvals for edited varieties, nano-formulation trial protocols, and AI surveillance data governance. Regulatory evolution will accelerate laboratory-to-farm technology transfer, supporting Vision 2030’s agricultural transformation goals.

### 8.11 Priority actions for plant virology framework

To translate strategic intent into operational impact, five evidence-based actions should be prioritized. First, a MEWA-led Saudi Plant Virus Database should integrate historical records with real-time surveillance to support rapid decision-making. Second, phased deployment of HTS-based national surveillance should target high-value, water-intensive crops to maximize return on investment. Third, dedicated funding is required for vector genomics and climate-linked epidemic forecasting to anticipate emergence risks. Fourth, a national seed health and biosecurity framework incorporating molecular certification is essential to reduce introductions. Finally, sustained capacity building in plant virology, bioinformatics, AI-driven monitoring, and regulatory science will ensure long-term system resilience and policy readiness.

## 9 Conclusion

Plant virology in Saudi Arabia has advanced from symptom-based diagnostics in the 1950s to molecular discovery, identifying over 81 viruses infecting 46 major crops, with +ssRNA viruses predominating. These viruses pose significant threats to economically important crops such as tomato, alfalfa, and cucurbits. However, persistent challenges from viruses like AMV, TYLCV, WmCSV, ToBRFV, and ZYMV—compounded by climate variability, vector expansion, and global trade—continue to jeopardize food security and sustainability goals under Vision 2030. To mitigate these risks, Saudi Arabia must channel strategic investments into advanced virus surveillance systems based on HTS, CRISPR, and AI platforms that enable real-time detection and early warning of viral outbreaks. Strengthened inter-institutional collaboration among KSU, KFU, KAUST, and MEWA is vital to unify national research efforts, standardize diagnostic protocols, and facilitate data sharing through a Saudi Plant Virus Database. Translating these scientific advancements into practice requires developing virus-resistant cultivars, expanding local bioinformatics capacity, and promoting IPM strategies that combine biotechnology, ecological regulation, and sustainable water use. By aligning technological innovation with collaborative governance and translational research, Saudi Arabia can transform plant virology into a cornerstone of its sustainable agriculture agenda—fortifying food security, reducing import dependence, and cementing its position as a pioneering hub for arid-zone plant health research.

## 10 Future perspectives

Saudi Arabia’s plant virus management roadmap should balance innovation with implementation feasibility. Initial priority must focus on strengthening existing virology networks by upgrading regional laboratories with standardized molecular tools and training programs. Then, targeted deployment of HTS surveillance in core agricultural areas, alongside the development of virus-resistant germplasm through public-private partnerships with established seed companies. Lastly, integration of advanced technologies (CRISPR, AI modeling) should be pursued subsequent to successful pilot studies and regulatory approval. Critical success factors include: (1) consolidating research efforts under a single MEWA-coordinated program rather than dispersed institutional initiatives, (2) prioritizing water-efficient resistant varieties over broad-spectrum surveillance, aligning with Vision 2030’s water security goals, and (3) focusing international collaboration on technology transfer rather than basic research. Collectively, this phased and coordinated approach would address current gaps and strengthen plant health systems in support of Vision 2030 goals.
